# Hyperactivation of NF-κB via the MEK signaling is indispensable for the inhibitory effect of cAMP on DNA damage-induced cell death

**DOI:** 10.1186/1476-4598-10-45

**Published:** 2011-04-21

**Authors:** Martine M Kloster, Elin H Naderi, Harald Carlsen, Heidi K Blomhoff, Soheil Naderi

**Affiliations:** 1 Department of Biochemistry, Institute of Basic Medical Sciences, University of Oslo, PO Box 1112 - Blindern, N-0317 Oslo, Norway; 2 Department of Nutrition, Institute of Basic Medical Sciences, University of Oslo, PO Box 1112 - Blindern, N-0317 Oslo, Norway; 3 Faculty of Health Sciences, Oslo University College, PO Box 4, St. Olavs Plass, N-0130, Oslo, Norway

## Abstract

With cAMP signaling having a profound inhibitory effect on DNA damage-induced apoptosis in B-cell precursor acute lymphoblastic leukemia (BCP-ALL) cells, understanding how this signaling pathway affects the survival capacity of the cell has important implications for cancer therapy. We have recently shown that p53 is critical for the inhibitory effect of cAMP on genotoxic agents-mediated apoptosis in BCP-ALLs. Here, we show that elevation of cAMP levels in cells exposed to DNA damage enhances the nuclear translocation and DNA binding of NF-κB by accelerating the phosphorylation of IKKβ and thereby phosphorylation and degradation of IκBα. Furthermore, we show that the ability of cAMP to potentiate the ionizing radiation-induced activation of NF-κB requires the activity of MEK. Importantly, pharmacological or genetic ablation of NF-κB reversed the inhibitory effect of cAMP on DNA damage-induced apoptosis, demonstrating that, in addition to p53, cAMP relies on the activity of NF-κB to provide cells with a survival advantage in the face of DNA damage. Collectively, our results uncover a novel and important interaction between the cAMP and NF-κB pathways that may have implications for the targeted treatment of lymphoid malignancies, such as BCP-ALL, in which aberrant NF-κB activity functions as a driving force for treatment resistance.

## Background

Activation of apoptosis in tumor cells is essential for the ability of cancer therapeutic drugs, such as genotoxic agents, to elicit a successful antineoplastic response [1,2]. Importantly, the apoptotic process in cancer cells is often compromised, enabling these cells to resist the cytotoxic effect of antitumor drugs and thus leading to the emergence of drug-resistant malignancies [3-5]. The ability of genotoxic agents to induce apoptosis in the target cancer cells is primarily influenced by the activity of two key signaling networks, the nuclear factor-κB (NF-κB) and p53 pathways [6,7]. NF-κB is a dimeric transcription factor that in the resting state is sequestered in the cytoplasm through its association with one of the inhibitory κB (IκB) proteins [8]. In response to DNA damage, the IκB kinase (IKK) complex phosphorylates IκBα at S32 and S36, an event that marks IκBα for ubiquitination and proteasomal degradation, thus allowing the NF-κB complex (p50/p65) to translocate to nucleus where it binds DNA and regulates the expression of a variety of genes, including antiapoptotic genes [7,9]. Consistent with this prosurvival function of NF-κB, inhibition of NF-κB activation has been shown to improve the apoptotic response of cells to cancer therapeutics [10]. Furthermore, the constitutive and deregulated activation of NF-κB found in many solid tumors as well as hematological malignancies is believed to promote cell survival and confer treatment resistance [9,11,12].

The transcription factor p53 is a tumor suppressor protein that is stabilized and activated in response to various types of cellular stress, including DNA damage [13,14]. This results in transactivation of a number of downstream genes whose products induce cell cycle arrest or apoptosis depending on the cell type and the nature of stress. For instance, lymphoid cells readily undergo p53-dependent apoptosis in response to DNA damage [15]. The inability to induce p53 or loss of normal p53 function is thought to facilitate cancer initiation and progression and to increase the survival potential of the cell in response to anticancer treatment.

In contrast to most carcinomas, the incidence of p53 mutations in hematological malignancies is notably low [16-18]. This indicates the involvement of other mechanisms that impinge on p53 and prevent its apoptosis-inducing effect. Based on our results in a recent study [19], we proposed cAMP signaling to be one such mechanism. We showed that activation of cAMP signaling in primary B-cell precursor acute lymphoblastic leukemia (BCP-ALL) blasts as well as BCP-ALL-derived cell lines inhibited the accumulation of p53 and protected the cells from DNA damage-induced apoptosis.

Given that the fate of cells exposed to DNA damage depends on the balance between the NF-κB-induced prosurvival signal and the p53-activated proapoptotic program [4], we wished to investigate whether NF-κB, in addition to p53, plays a role in the ability of cAMP to diminish the apoptotic response of BCP-ALL cells to DNA damage. Here, we show that cAMP potentiates the induction of NF-κB by DNA damage. Furthermore, we show that attenuation of NF-κB activity reverses the inhibitory effect of cAMP on DNA damage-induced apoptosis. Importantly, our results indicate a critical role for MEK signaling in mediating the potentiating effect of cAMP on DNA damage-induced NF-κB activation. Based on these results, we conclude that cAMP, through inhibition of p53 accumulation and simultaneous potentiation of NF-κB activity, renders cells resistant to the apoptosis-inducing effect of DNA damage. Thus, the potential use of NF-κB modulators may prove beneficial in treatment of cancers in which aberrant activation of cAMP signaling endows the cells with a prosurvival advantage.

## Results

### Alleviation of NF-κB activity reverses the inhibitory effect of cAMP on IR-induced cell death

**Figure 1:**
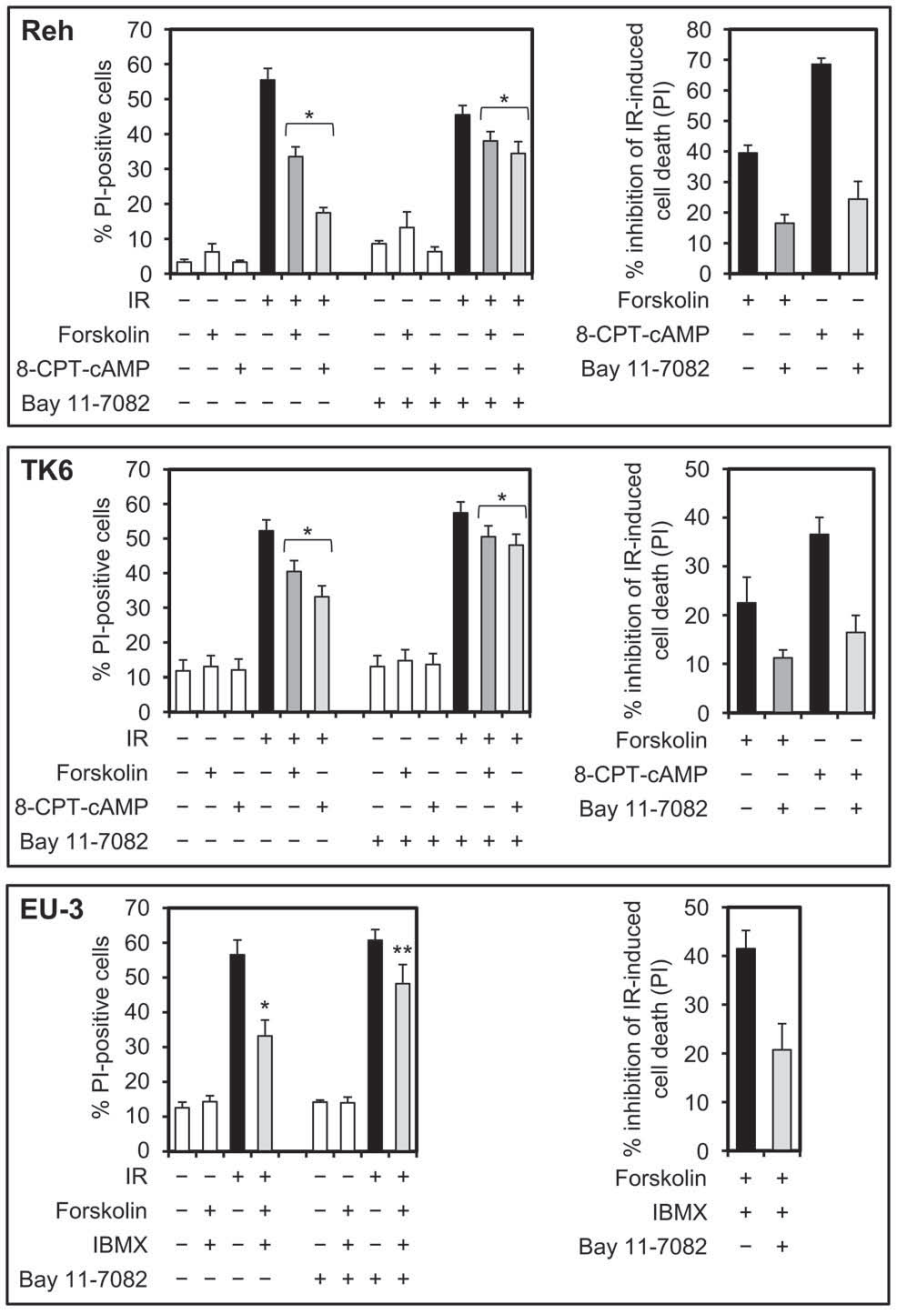
**Pharmacological attenuation of NF-κB activity alleviates the inhibitory effect of forskolin or 8-CPT-cAMP on IR-induced cell death**. Cells were cultured in the absence or presence of 5 μM Bay 11-7082 alone (Reh and TK6) or together with 400 μM IBMX (EU-3) for 90 min before treatment with or without 80 μM forskolin (Reh and TK6), 100 μM forskolin (EU-3) or 200 μM 8-CPT-cAMP for 30 min. Cells were then exposed to 10 Gy IR, harvested after 12 h (EU-3) or 20 h (Reh and TK6) and analyzed for PI uptake by FACS (n = 4). *p < .01, **p < .03 relative to cells treated with IR only. The histograms in the right panel depict percent inhibition of IR-induced cell death by forskolin, 8-CPT-cAMP or forskolin and IBMX in the presence or absence of Bay 11-7082.

**Figure 2:**
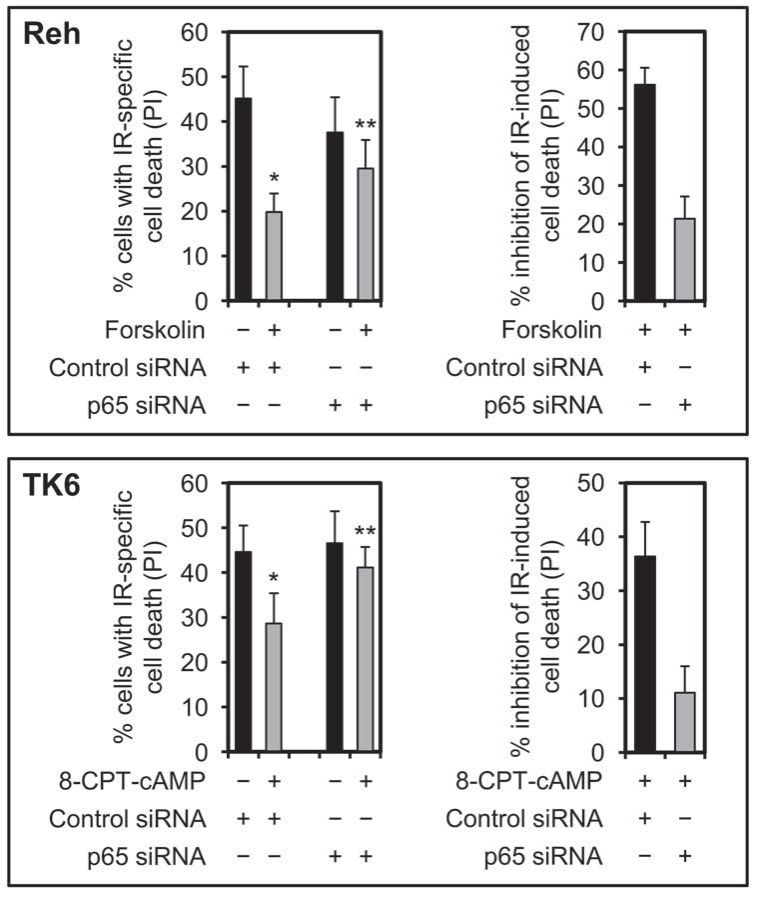
**Knockdown of p65 relieves the inhibitory effect of forskolin or 8-CPT-cAMP on IR-induced cell death**. Cells transfected with control siRNA or p65 siRNA were treated with or without forskolin (80 μM) or 8-CPT-cAMP (200 μM) for 30 min before exposure to IR (10 Gy). After 20 h, cells were analyzed for PI uptake by FACS (n = 4). The *p *values were calculated relative to cells treated with IR only: Reh, *p < .01, **p < .04. TK6, *p < .03, **p < .05. The histograms in the right panel depict percent inhibition of IR-induced cell death by forskolin or 8-CPT-cAMP in cells transfected with control siRNA or p65 siRNA.

In our recent study, we showed that stimulation of cAMP signaling inhibits DNA damage-induced accumulation of p53 and apoptosis in BCP-ALL cells [19]. Given the observations that DNA damage, in addition to induction of p53, also engages the prosurvival NF-κB pathway, we wished to examine whether NF-κB plays a role in cAMP-mediated inhibition of DNA damage-induced cell death. To do so, we used the BCP-ALL cell lines Reh and EU-3 and the lymphoblastoid cell line TK6, all of which express wt p53, and examined the inhibitory effect of cAMP signaling on DNA damage-induced cell death in the presence of the NF-κB inhibitor Bay 11-7082. As previously demonstrated, induction of cAMP levels by forskolin, an activator of adenylyl cyclase, 8-CPT-cAMP, a membrane-permeable analog of cAMP, or a combination of forskolin and the phosphodiesterase inhibitor, IBMX, inhibited the IR-induced cell death (Figure 1). Interestingly, whereas treatment of cells with Bay 11-7082 had marginal effect on cell death induced by IR alone, it markedly alleviated the inhibitory effect of cAMP-elevating agents on IR-induced cell death in all three cell types. To further confirm this observation, we performed siRNA-mediated knockdown of the p65 subunit of NF-κB in Reh and TK6 cells and examined the effect of elevation of cAMP levels on DNA damage-induced cell death. As shown in Figure 2, knockdown of p65 by siRNA alleviated the inhibitory effect of cAMP-elevating agents on IR-induced cell death in Reh and TK6 cells. Taken together, these results indicate that NF-κB is required for the inhibitory effect of cAMP on DNA damage-induced cell death.

### cAMP enhances IR-induced phoshorylation and degradation of IκBα and subsequent activation of NF-κB

**Figure 3:**
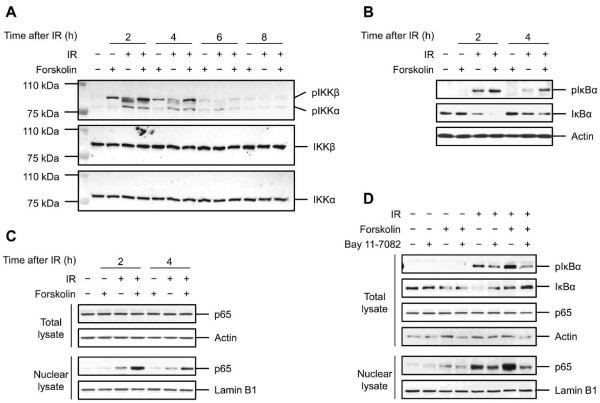
**cAMP enahnaces the IR-induced phosphorylation of IKKβ and nuclear translocation of p65**. (A) Reh cells were treated with or without forskolin (80 μM) for 30 min before irradiation (10 Gy). Cells were harvested at the indicated times and subjected to Western blot analysis with the indicated antibodies. The figure shows 1 representative blot of 4 experiments. (B) Reh cells were treated as in A, harvested at the indicated times and subjected to immunoblot analysis with the indicated antibodies. The figure shows 1 representative blot of 4 experiments. (C) Reh cells were treated as in A. One half of each sample was used for preparation of total cell lysate and the other half was subjected to cellular fractionation to obtain nuclear lysate. The lysates were then analyzed by immunoblotting with the indicated antibodies. The figure shows 1 representative experiment of 3. (D) Reh cells were cultured in the absence or presence of Bay 11-7082 (5 μM) for 90 min before treatment with or without forskolin (80 μM) for 30 min. Cells were then exposed to 10 Gy IR and harvested after 2 h. One half of each sample was used for preparation of total cell lysate and the other half was subjected to cellular fractionation to obtain nuclear lysate. The lysates were then analyzed by immunoblotting with the indicated antibodies. The figure shows 1 representative experiment of 3.

Given the observation that inhibition of NF-κB activity reverses the cAMP-mediated inhibition of DNA damage-induced cell death, we examined key events in the NF-κB pathway to assess whether this pathway is regulated in response to elevation of cAMP levels. Induction of NF-κB activity typically requires the activity of the IκB kinase (IKK) complex, consisting of IKKα, IKKβ kinases and the scaffold protein IKKγ/NEMO [8]. Upon phosphorylation of the activation loops of IKKα (S176/S180) and IKKβ (S177/S181), the IKK complex is activated and phosphorylates the NF-κB-bound IκB proteins, targeting them for proteasomal degradation and thus allowing NF-κB to translocate to nucleus where it binds to NF-κB target promoters and regulates their target genes [20-24]. To examine the effect of cAMP on the activity of IKK, Reh cells were exposed to IR in the absence or presence of forskolin, collected at regular intervals for a total of 8 h, and analyzed by Western blotting with antibodies specific for phosphorylated IKKα (S176/S181) and IKKβ (S177/S181). As shown in Figure 3A, exposure of cells to IR led to phosphorylation of IKKα and IKKβ within 2 h. By 4 h after IR, phosphorylation of IKKα and IKKβ declined slightly and continued to decrease thereafter so that by 8 h postirradiation it had reached the basal levels found in untreated cells. Interestingly, forskolin alone led to phosphorylation of IKKβ. Furthermore, exposure of cells to IR in the presence of forskolin potentiated the IR-induced phosphorylation of IKKβ. In both cases, the kinetics of forskolin-induced phosphorylation of IKKβ correlated closely with phosphorylation of IKKβ induced by IR alone.

Having identified cAMP as an inducer of IKKβ phosphorylation, we proceeded to examine the effect of cAMP on phosphorylation and degradation of IκBα. To this end, Reh cells were treated with IR in the absence or presence of forskolin, harvested at 2 and 4 h postirradiation and subjected to immunoblot analysis using an anti-phospho-IκBα antibody and an antibody recognizing nonphosphorylated IκBα. In parallel with kinetics of phosphorylation of IKKα and IKKβ, phosphorylation and degradation of IκBα was induced by 2 h after exposure of cells to IR before declining at 4 h postirradiation (Figure 3B). Furthermore, exposure of cells to forskolin potentiated the effect of IR on IκBα phosphorylation and degradation with similar kinetics. Interestingly, although exposure of cells to forskolin alone increased phosphorylation of IKKβ, it failed to induce phosphorylation and degradation of IκBα. The ability of forskolin to potentiate the IR-induced phosphorylation-dependent degradation of IκBα suggested that forskolin would enhance the IR-mediated nuclear accumulation of NF-κB. To examine this notion, Reh cells that were exposed to IR in the absence or presence of forskolin were subjected to subcellular fractionation and the nuclear fraction was analyzed by immunoblotting with antibodies against the p65 subunit of NF-κB. As shown in Figure 3C, in conformity with kinetics of IκBα degradation, the expression of nuclear p65 was induced by 2 h after exposure of cells to IR before declining slightly at 4 h postirradiation. Notably, treatment of cells with forskolin had a marked enhancing effect on the IR-induced nuclear accumulation of p65 at both time points.

Next, we wished to examine whether the enhancing effect of cAMP on IR-mediated activation of NF-κB pathway requires the activity of IKK kinase, the enzyme responsible for phosphorylation of IκBα and thus induction of NF-κB. To this end, we examined the effect of Bay 11-7082 on forskolin-mediated regulation of IR-induced phosphorylation and degradation of IκBα as well as nuclear translocation of p65. Bay 11-7082 is an inhibitor of IKK kinase and attenuates the phosphorylation and subsequent degradation of the NF-κB inhibitor, IκBα. As expected, Bay 11-7082 inhibited the IR-induced phosphorylation and degradation of IκBα, and thus attenuated the translocation of p65 into the nucleus (Figure 3D). Importantly, in cells exposed to IR in the presence of forskolin, Bay 11-7082 inhibited the phosphorylation and degradation of IκBα as well as the nuclear translocation of p65 to levels similar to those found in cells that were treated with only IR in the presence of Bay 11-7082. Thus, the stimulatory effect of cAMP on IR-induced activation of NF-κB pathway depends on IKK kinase activity.

**Figure 4:**
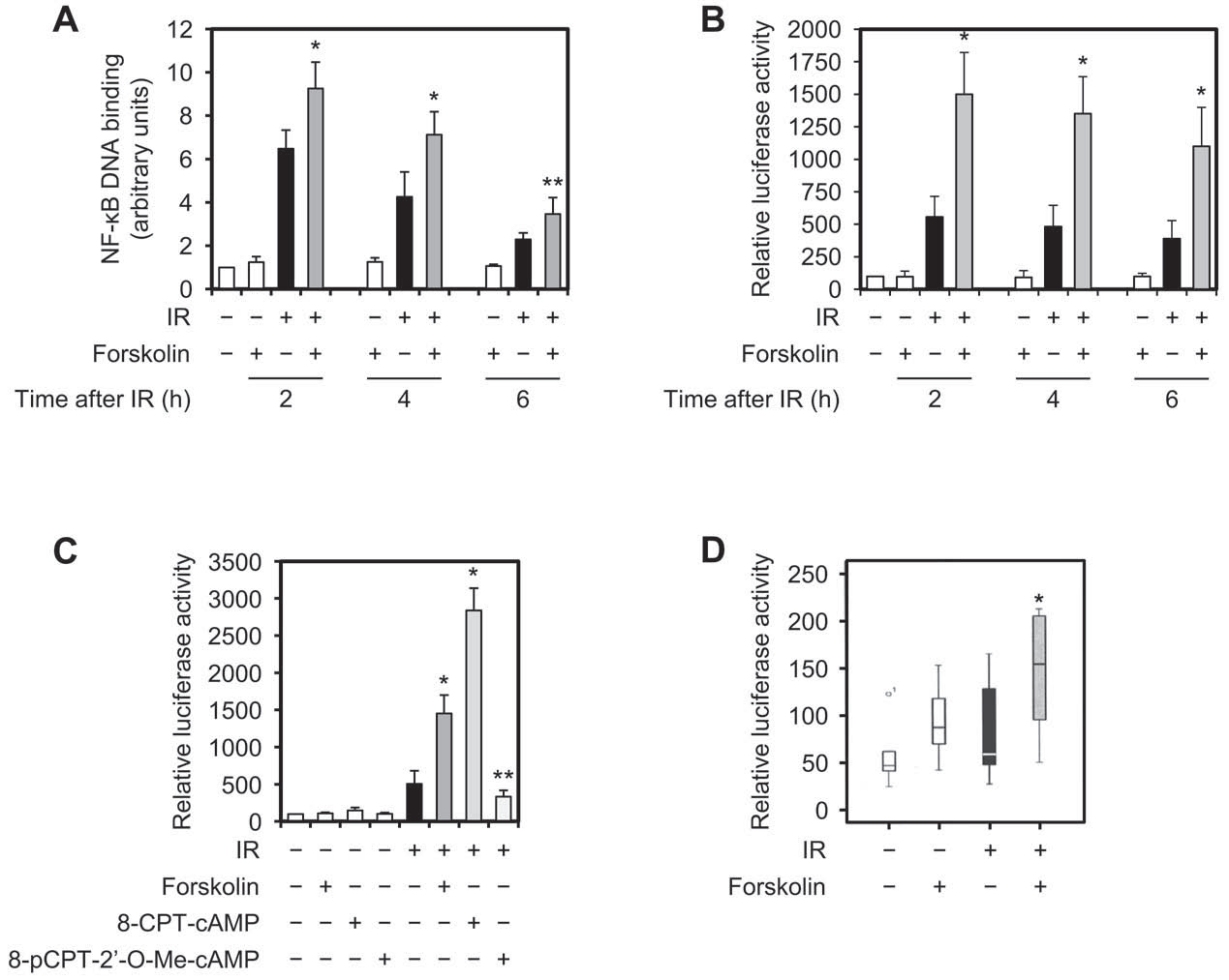
**Potentiation of NF-κB DNA binding and transcriptional activity by cAMP**. (A) Reh cells were treated with or without forskolin (80 μM) for 30 min before irradiation (10 Gy). Cells were harvested at the indicated times after IR and nuclear extracts were prepared. The binding activity of p65 in the nuclear extracts was then determined by the p65 TransAM assay (n = 4). *p < .01, **p < .05 relative to cells treated with IR only. (B) Reh cells were transfected with a plasmid encoding the luciferase gene under 3 repeats of a NF-κB consensus binding site. After 24 h, cells were cultured in the presence or absence of forskolin (80 μM) for 30 min before exposure to IR (10 Gy). At the indicated times after IR, cells were harvested and the luciferase activity was measured as described in Materials and methods (n = 4). *P < .01 relative to cells treated with IR only. (C) Reh cells were transfected as in B. After 24 h, cells were cultured in the presence or absence of forskolin (80 μM), 8-CPT-cAMP (200 μM) or 8-pCPT-2'-O-Me-cAMP (400 μM) for 30 min before exposure to IR (10 Gy). Cells were harvested at 4 h after IR and luciferase activity was measured as described in Materials and methods (n = 4). *P < .01, **P < .05 relative to cells treated with IR only. (D) Freshly isolated splenocytes from 3 × κB-luc transgenic mice were cultured in the presence or absence of forskolin (80 μM) for 30 min before exposure to IR (10 Gy). After 2 h, luciferase activity was measured as described in Materials and Methods. The boxes show the median, upper and lower quartile, and the whiskers show the range of values (n = 9, *P < .05 by Wilcoxon signed rank test when compared with cells treated with IR only). The outlier value (○) stems from one mouse.

To determine the potential enhancing effect of cAMP on IR-induced NF-κB DNA binding, we used an ELISA-based assay to measure IR-mediated DNA binding in the absence or presence of forskolin. Figure 4A shows that exposure of Reh cells to IR led to a robust increase in the amount of NF-κB bound to the consensus site oligonucleotides by 2 h. Thereafter, the NF-κB DNA binding activity decreased gradually so that by 6 h post-IR it had reached to a level slightly above that found in untreated cells. Importantly, exposure of cells to forskolin significantly enhanced the IR-induced DNA binding of NF-κB at all time points.

Next, we wished to examine whether cAMP affected the NF-κB-dependent gene transcription. We transiently transfected Reh cells with an NF-κB-dependent luciferase reporter construct and examined them by luciferase assay after exposure to IR in the presence or absence of forskolin. As shown in Figure 4B, the NF-κB luciferase reporter activity was relatively low in untreated cells or cells that were treated with forskolin alone. Exposure of cells to IR increased the NF-κB promoter activity 5 fold within 2 h. The transcriptional activity of NF-κB then decreased gradually so that by 6 h after IR, it was induced 4 fold compared to untreated cells. Notably, pretreatment of cells with forskolin had a profound potentiating effect on the IR-induced NF-κB-dependent transcription at all time points.

Two major downstream targets of cAMP are protein kinase A (PKA) and exchange protein directly activated by cAMP (Epac) [25-27]. To examine whether the enhancing effect of cAMP on NF-κB activity is mediated through PKA or Epac, Reh cells that were transfected with an NF-κB-dependent luciferase reporter construct were exposed to IR in the absence or presence of 8-CPT-cAMP or 8-pCPT-2'-O-Me-cAMP and then examined for NF-κB luciferase reporter activity. 8-CPT-cAMP is an activator of both PKA and Epac, whereas 8-pCPT-2'-O-Me-cAMP is a potent and specific agonist of Epac with no effect on PKA activity [28]. As can be seen in Figure 4C, pretreatment of Reh cells with 8-CPT-cAMP had a robust potentiating effect on IR-induced NF-κB activity. In contrast, exposure of cells to a concentration of 8-pCPT-2'-O-Me-cAMP as high as 400 μM did not enhance the NF-κB transcriptional activity in IR-treated cells, indicating that cAMP potentiates the IR-induced NF-κB activity in a PKA-dependent manner.

Finally, to confirm that the enhancing effect of cAMP on the NF-κB activity also occurs in normal cells, we used splenocytes isolated from 3 × κB-luc transgenic mice [29], and examined them for luciferase activity after exposure to IR in the absence or presence of forskolin. Similar to the results obtained with Reh cells, treatment of splenocytes with IR led to an increase in luciferase activity within 2 h (Figure 4D). Furthermore, pretreatment of these cells with forskolin significantly enhanced the IR-induced NF-κB activity.

### MEK signaling is required for cAMP-mediated activation of NF-κB

**Figure 5:**
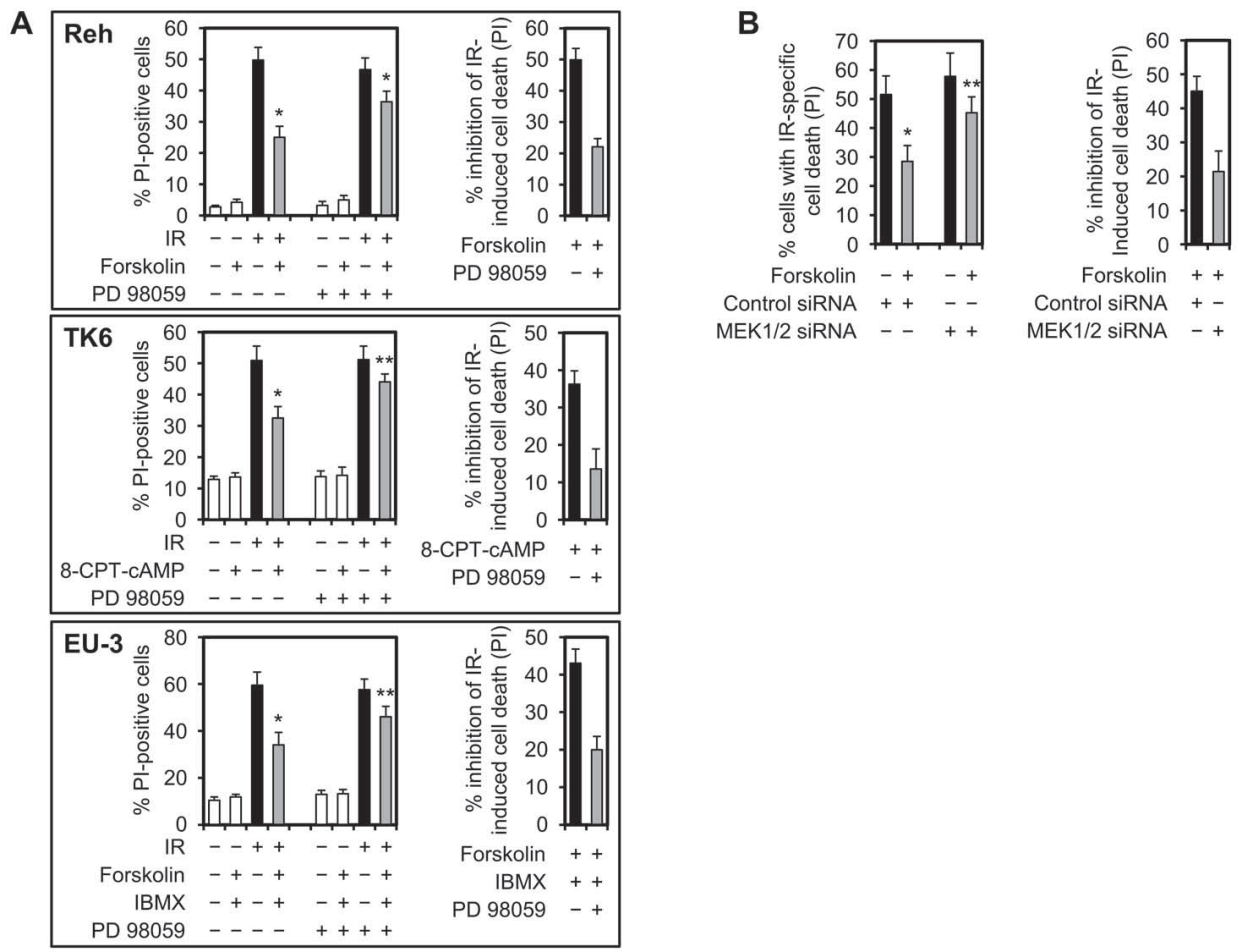
**Inhibition of MEK signaling alleviates the inhibitory effect of cAMP on IR-induced cell death**. (A) Cells were cultured in the absence or presence of 50 μM PD 98059 alone (Reh and TK6) or together with 400 μM IBMX (EU-3) for 45 min before treatment with or without 80 μM forskolin (Reh), 200 μM 8-CPT-cAMP (TK6) or 100 μM forskolin (EU-3) for 30 min. Cells were then exposed to 10 Gy IR, harvested after 12 h (EU-3) or 20 h (Reh and TK6) and analyzed for PI uptake by FACS (n = 4). The *p *values were calculated relative to cells treated with IR only: Reh, *p < .02. TK6, *p < .01, **p < .03. EU-3, *p < .01, **p < .04. The histograms in the right panel depict percent inhibition of IR-induced cell death by forskolin, 8-CPT-cAMP or forskolin and IBMX in the presence or absence of PD 98059. (B) Reh cells were transfected with control siRNA or siRNAs against MEK1 and MEK2. Cells were then treated with or without forskolin (80 μM) for 30 min before exposure to IR (10 Gy). After 20 h, cells were analyzed for PI uptake by FACS (n = 4). *p < .01, **P < .03 relative to cells treated with IR only. The histogram in the right panel depicts percent inhibition of IR-induced cell death by forskolin in cells transfected with control siRNA or MEK1 and MEK2 siRNAs.

**Figure 6:**
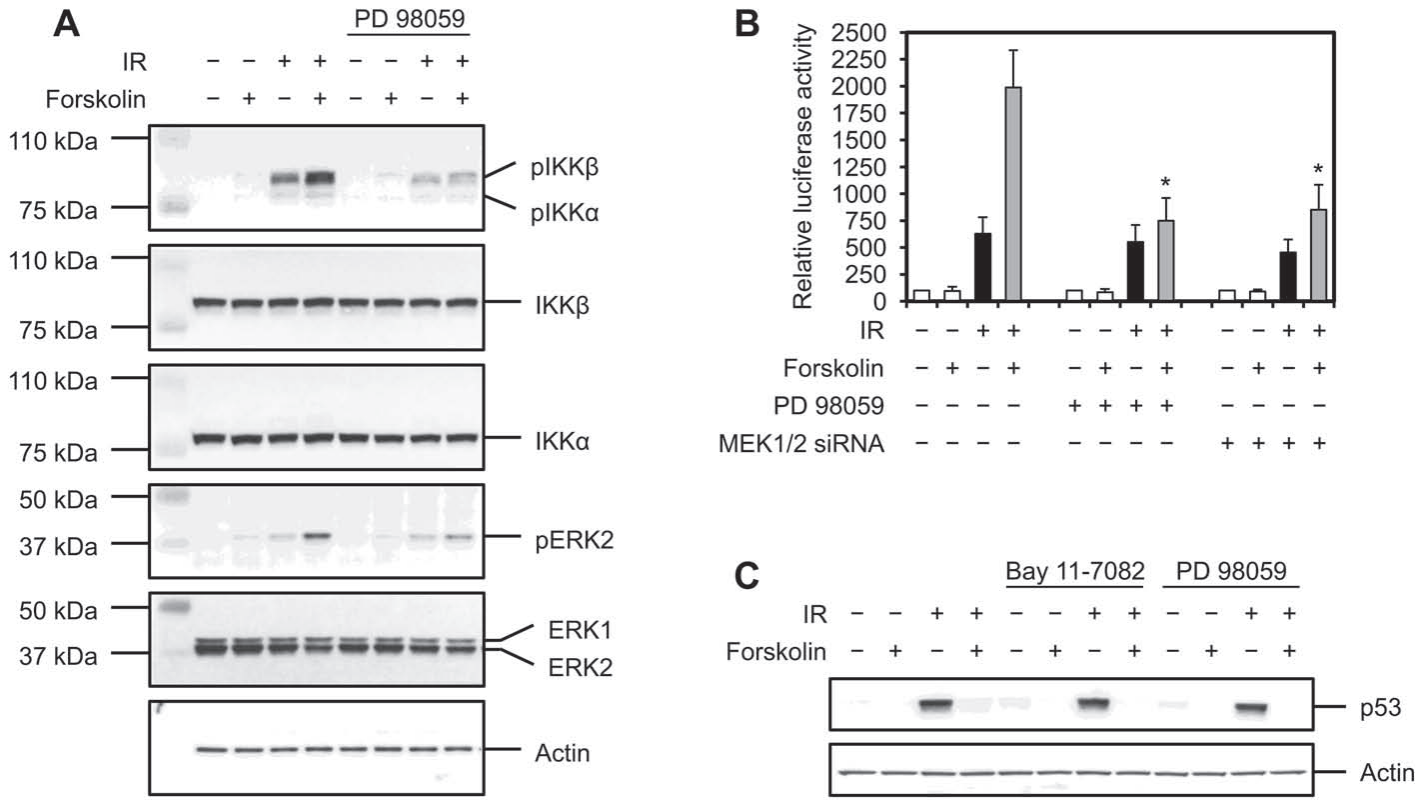
**MEK signaling is required for cAMP to affect the IR-induced NF-κB activation**. (A) Reh cells were cultured in the presence or absence of 50 μM PD 98059 for 45 min before treatment with or without forskolin (80 μM) for 30 min. cells were then exposed to IR (10 Gy), harvested at 2 h postirradiation and subjected to immunoblot analysis with the indicated antibodies. The figure shows 1 representative blot of 3 experiments. (B) Reh cells were transfected with a plasmid encoding the luciferase gene under 3 repeats of a NF-κB consensus binding site or left untreated. After 24 h, cells were treated as in A, harvested at 4 h postirradiation and analyzed for luciferase activity as described in Materials and methods (n = 4). *p < .02 relative to cells treated with IR only. (C) Reh cells were cultured in the absence or presence of 5 μM Bay 11-7082 for 90 min or 50 μM PD 98059 for 45 min before treatment with or without forskolin (80 μM) for 30 min. Cells were then exposed to IR (10 Gy), harvested after 4 h and subjected to Western blot analysis with the indicated antibodies. The figure shows 1 representative blot of 3 experiments.

The involvement of ATM in activation of NF-κB following DNA damage raises the possibility that cAMP, by enhancing the activity of ATM, could potentiate the IR-induced activation of NF-κB. However, our finding that cAMP does not affect the activity of ATM after DNA damage argues against this notion [19]. Therefore, to assess the mechanism underlying the potentiating effect of cAMP on IR-mediated activation of NF-κB, we directed our attention to molecular events downstream of ATM. The MEK signaling is coupled to NF-κB in a number of stress-induced responses [30,31]. Furthermore, the MEK pathway has been reported to be activated in an ATM-dependant manner and induce the activity of IKK, thereby leading to activation of NF-κB [32,33]. Therefore, we chose to investigate the potential role of this pathway in mediating the enhancing effect of cAMP on NF-κB activation and cell survival. First, we determined the effects of MEK inhibition on the ability of cAMP to inhibit the DNA damage-induced cell death. Treatment of Reh, TK6 or EU-3 cells with the MEK inhibitor PD 98059 alone did not have an appreciable effect on IR-induced cell death (Figure 5A). Notably, pretreatment of cells with PD 98059 attenuated the inhibitory effect of elevated levels of cAMP on IR-induced cell death in all three cell types. To further confirm this result, we transfected Reh cells with siRNAs directed against MEK1 and MEK2 and examined them for cell death following exposure to IR in the absence or presence of forskolin. As shown in Figure 5B, whereas disruption of MEK1 and MEK2 genes expression did not inhibit the IR-induced cell death, it alleviated the ability of forskolin to attenuate cell death induced by IR. Next, we wished to examine the effect of MEK1 and MEK2 inhibition on the ability of cAMP to enhance the IR-mediated induction of IKK phosphorylation. To do so, Reh cells that were treated with PD 98059 were exposed to IR in the absence or presence of forskolin and harvested at 2 h postirradiation for examination of MEK1 and MEK2 phosphorylation by Western blotting. Whereas treatment of cells with PD 98059 had a slight inhibitory effect on IKKβ phosphorylation induced by IR alone, it profoundly attenuated the enhancing effect of forskolin on IR-induced phosphorylation of IKKβ (Figure 6A). Importantly, enhancement of IKKβ phosphorylation by forskolin in IR-treated cells correlated with potentiation of ERK2 phosphorylation. Because ERKs are predominant downstream targets of MEK [34], this result suggests the notion that cAMP enhances the activity of MEK.

To assess the role of MEK signaling in cAMP-mediated enhancement of NF-κB activity following DNA damage, MEK activity in Reh cells that were transfected with an NF-κB-dependent luciferase reporter construct was inhibited by either treatment of cells with PD 98059 or by RNA interference. Cells were then examined by luciferase assay after exposure to IR in the presence or absence of forskolin. As shown in Figure 6B, whereas disruption of MEK signaling with either PD 98059 or MEK1 and MEK2 siRNAs slightly decreased the IR-induced NF-κB promoter activity, it substantially inhibited the potentiating effect of cAMP on IR-mediated NF-κB-dependent transcription.

Finally, to rule out the possibility that the reversal of the inhibitory effect of cAMP on IR-induced cell death through attenuation of MEK1 and MEK2 or NF-κB activities may be due to stabilization of p53, we examined the expression of p53 in cells that were treated with PD 98059 or Bay 11-7082. As shown in Figure 6C, pretreatment of Reh cells with PD 98059 or Bay 11-7082 had no effect on the basal level of p53 protein. The inability of Bay 11-7082 to increase the level of p53 is in contrast with previous studies showing that IKKβ decreases the stability of p53 protein [35,36]. Furthermore, inhibition of MEK1 and MEK2 or NF-κB did not affect the ability of forskolin to attenuate the IR-mediated accumulation of p53. Collectively, these results indicate that MEK-NF-κB signaling axis plays an important p53-independent role in the inhibitory effect of cAMP on DNA damage-induced cell death.

### cAMP induces the expression of survivin in IR-treated cells in an NF-κB-dependent manner

**Figure 7:**
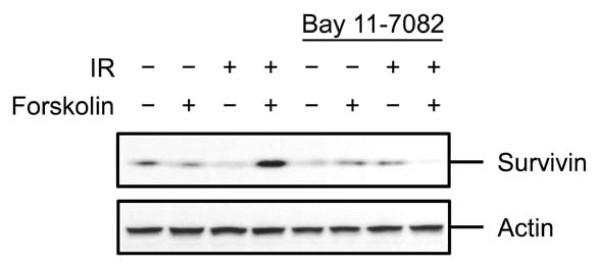
**cAMP-induced expression of survivin in IR-treated cells**. Reh cells were cultured in the absence or presence of 5 μM Bay 11-7082 for 90 min before treatment with or without forskolin (80 μM) for 30 min. Cells were then exposed to IR (10 Gy), harvested after 12 h and subjected to Western blot analysis with the indicated antibodies. The figure shows 1 representative blot of 3 experiments.

To address the mechanism by which cAMP-induced hyperactivation of NF-κB mediates the inhibitory effect of cAMP on DNA damage-induced cell death, we proceeded to examine the expression of a number of NF-κB-regulated antiapoptotic proteins, such as Bcl-xL, c-IAP1, MCL-1, XIAP and survivin [37]. To this end, Reh cells that were treated with or without Bay 11-7082 were exposed to IR in the absence or presence of forskolin and harvested at 12 h postirradiation for examination of by Western blotting. Exposure of cells to IR alone or to IR in the presence of forskolin had no effect on the expression of Bcl-xL, c-IAP1, MCL-1 or XIAP (data not shown). Interestingly, whereas exposure of Reh cells to IR slightly reduced the level of survivin, pretreatment of cells with forskolin substantially induced the expression of survivin (Figure 7). Notably, this effect of forskolin was attenuated by treatment of cells with Bay 11-7082, indicating that cAMP-induced expression of survivin in DNA-damaged cells is mediated by NF-κB. Given the ability of survivin to inhibit cell death [38], these results suggest that at least one of the mechanisms by which NF-κB mediates the inhibitory effect of cAMP on DNA damage-induced cells death is through its capacity to induce the expression of survivin.

## Discussion

**Figure 8:**
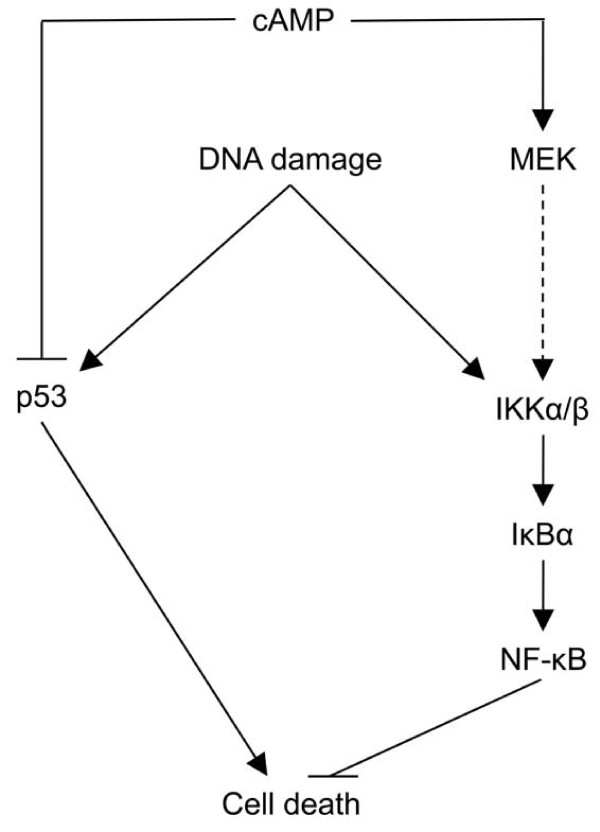
**Model depicting how cAMP potentiates the activation of NF-κB by DNA damage and modulates cell death**.

Stress signals, such as DNA damage, activate both the proapoptotic p53 and the prosurvival NF-κB pathways [7,6]. Thus, cell fate after DNA damage is determined by the outcome of the competition between these two antagonistic signaling pathways [4]. Therefore, it is not surprising that tumor cells utilize mechanisms to block p53 induction and induce NF-κB activation in order to avoid genotoxic-mediated killing. We believe that cAMP signaling represents such a mechanism. In this study we have identified NF-κB as a target of cAMP signaling in cellular response to DNA damage. We report that activation of the cAMP signal transduction pathway enhances the DNA damage-induced phosphorylation and activation of IKKβ, thereby facilitating the IKK-mediated phosphorylation and degradation of IkBα, an event that augments the activity of NF-κB in cells afflicted with DNA damage. Based on these findings together with our previous result demonstrating the inhibitory effect of cAMP on p53 accumulation [19], we propose a model in which activation of cAMP signaling in B cells exerts a protective effect against DNA damage-induced apoptosis by simultaneously downregulating the proaoptotic p53 protein and enhancing the activity of the prosurvival NF-κB protein (Figure 8). The ability of cAMP to affect both of these two antagonistic signaling pathways in order to endow the cell with a survival advantage may be of particular importance in tumors that retain wt p53. It may be suggested that acquisition of maximal protection against DNA damage in such tumors requires not only abrogation of the p53 function but also induction of the NF-κB activity.

In line with our finding that the cAMP-mediated inhibition of DNA damage-induced cell death depends on the ability of cAMP to hyperactivate NF-κB, during the final phase of the preparation of this manuscript, Safa et al. reported that elevation of cAMP in doxorubicin-exposed Nalm-6 cells induced the activity of NF-κB [39]. Furthermore, these authors showed that NF-κB activity contributes to the ability of cAMP to inhibit cell death in doxorubicin-treated Nalm-6 cells. However, in contrast to our findings showing that elevation of cAMP in DNA-damaged Reh cells induces the expression of survivin without affecting the levels of Bcl-2 or XIAP (for Bcl-2 see [19]), Safa et al. showed that cAMP increases the expression of Bcl-2 and XIAP in doxorubicin-treated Nalm-6 cells.

Given the importance of NF-κB activity in oncogenesis as well as its contribution to suppression of the apoptotic potential in cancer therapy [9,12,40], we also examined the mechanism by which cAMP signaling enhances the DNA damage-induced NF-κB activation. Inactive NF-κB dimers are sequestered in the cytoplasm in association with IκB proteins [8]. Following DNA damage, activated ATM interacts with NEMO in the nucleus and the resulting ATM/NEMO complex translocates to cytoplasm where it activates IKK complex, leading to phosphorylation and subsequent degradation of IκBα [7]. This event facilitates translocation of NF-κB into nucleus where it binds DNA and activates an antiapoptotic transcriptional program. Our observation that forskolin enhances the DNA damage-induced phosphorylation and degradation of IκBα indicated that cAMP positively regulates the IKK complex to induce NF-κB activation. This conclusion was further supported by the finding that inhibition of IKK activity by Bay 11-7082 alleviated the potentiating effect of cAMP on IκBα phosphorylation and degradation. cAMP could enhance the activity of NF-κB by modulating the ATM-NEMO axis. However, the inability of cAMP to affect the DNA damage-induced activation of ATM [19] suggested that cAMP enhanced NF-κB activity through an alternative mechanism. The finding that abrogation of MEK activity abolishes the ability of cAMP to potentiate the DNA damage-induced activity of NF-κB indicates that MEK signaling functions in such capacity. Importantly, disruption of MEK signaling had no appreciable effect on the activity of NF-κB in cells that were exposed to IR alone. This observation is in contradiction with the results of Panta et al [33] showing that inhibition of MEK results in downregulation of doxorubicin- or IR-mediated activation of NF-κB. Whereas the cells utilized by Panta et al were mainly of fibroblast origin, we have exclusively used cells of B lymphocyte lineage. Therefore, this discrepancy may be explained by the suggestion that the MEK pathway relays the DNA damage signal to NF-κB in a cell type-specific manner. Notwithstanding, our results show that, at least in BCP-ALL and lymphoblastoid cells, the MEK signaling engages the NF-κB pathway in DNA damaged cells only when cAMP signaling is activated.

The mechanism by which cAMP activates MEK signaling should also be addressed. It can be envisioned that stimulation of MEK phosphorylation and activation by cAMP can be achieved by (i) direct phosphorylation of MEK by PKA, or (ii) positive regulation of an activating event upstream of MEK proteins. The finding that MEK is not efficiently phosphorylated by PKA *in vitro *[41] diminishes the possibility that *in vivo *PKA activates MEK through direct phosphorylation. At present, we favor the second possibility in which cAMP induces the activity of a factor that is required for phosphorylation and activation of MEK. This mechanism is supported by the findings that cAMP stimulates MEK activity though activation of B-RAF pathway [42].

Degradation of IκBα and the resulting nuclear translocation of NF-κB and its binding to DNA are necessary but insufficient events for the induction of an NF-κB response. Covalent modifications of key residues of NF-κB are also crucial for its transcriptional activity downstream of IκBα [43]. Furthermore, these modifications are thought to determine the strength and duration of the NF-κB transcriptional response. For instance, inducible phosphorylation of p65 on S276 by PKA has been shown to promote its interaction with transcriptional coactivators p300 and CBP and thus enable NF-κB to activate gene transcription [44-46]. In the absence of antibodies specific for phosphorylated p65 on S276 [47], we can only speculate that elevation of cAMP in cells exposed to DNA-damaging agents most probably leads to phosphorylation of p65 on S276, thus enhancing the DNA damage-induced transcriptional activity of NF-κB. In such case, cAMP signaling would positively affect the activity of NF-κB at two levels: one involves enhancement of DNA damage-induced phosphorylation and degradation of IκBα, an event that positively regulates nuclear translocation of NF-κB. At the second level, cAMP, by amplifying the PKA-dependent phosphorylation of p65, stimulates the transcriptional activity of NF-κB.

Alterations in NF-κB activity is recognized as key pathological feature in various lymphoid malignancies [12]. Indeed, aberrant activity of NF-κB occurs in nearly all childhood ALL tumors [48], an event suggested to contribute to resistance of these cells to DNA damage. The credentials of cAMP as an antiapoptotic factor in BCP-ALL cells [19] and its ability to hyperactivate NF-κB lend further support to our notion that inhibitors of cAMP signaling pathway might prove beneficial in treatment of BCP-ALL tumors.

## Material and methods

### Reagents and antibodies

Forskolin and propidium iodide (PI) were obtained from Sigma-Aldrich. PD 98059 was purchased from Tocris Bioscience. Bay 11-7082 was obtained from Calbiochem. 8-CPT-cAMP and 8-pCPT-2'-O-Me-cAMP were from BioLog. Luciferase Assay system was from Promega. Antibodies against IκBα (#9242), phospho-IκBα (S32; #2859), p65 (#3034), IKKα (#2682), IKKβ (#2684), phospho-IKKα/β (S176/S180; #2697), ERK1/2 (#9102), and phosphor-ERK1/2 (T202/Y204; #9101) were from Cell Signaling Technology. Anti-actin (H196) and anti-Lamin B1 (H-90) were obtained from Santa Cruz Biotechnology.

### Cell cultures, radiation treatment and cell death analysis

Reh [49], EU-3 and TK6 were cultured as previously described [19]. For isolation of mice splenocytes, mice were sacrificed by cervical dislocation and spleens were removed and homogenized in a petri dish. Splenocytes were washed and adjusted to 2 × 10^6 ^cells/ml in RPMI supplemented with 10% heat-inactivated fetal bovine serum (Life Technologies), 2 mM glutamine, 125 U/ml penicillin, 125 μg/ml streptomycin (Gibco), and 50 μM β-mercaptoethanol (Sigma-Aldrich) at 37 °C in a humified incubator with 5% CO2. For treatment of cells with γ-radiation, cells were exposed to a 137Cs source at a dose rate of 4.3 Gy/min using a Gammacell irradiator from MSD Nordion. To analyze cell death, cells were incubated with PI (20 μg/ml) at room temperature for 10 min before examination for PI uptake by flow cytometry.

### Transfection and reporter gene assay

For siRNA transfection, Reh or TK6 cells (6 × 10^6^) were transfected with 16 pmol Signalsilence NF-κB p65 siRNA (6261; Cell signaling Technology) or stealth RNAi for human MEK1 and MEK2 (12935-025; Invitrogen) using the nucleofection solution R and the O-17 program (Reh) or solution V and the X-05 program (TK6) with a nucleofector device (Amaxa Biosciences). SignalSilence Control siRNA (6201; Cell Signaling technology) or control siRNA (12935-300; invitrogen) were used as controls for p65 and MEK1/2 siRNAs, respectively. Cells were then incubated for 24 h before further treatment. For reporter gene assay, Reh cells were cotransfected with 8 μg κB-luciferase plasmid and 4 μg β-galactosidase expression vector. 20 h after transfection, cells were subjected to further treatment. To prepare lysates, 100 μl reporter lysis buffer (Promega) was added to each sample, and the supernatant was collected after centrifugation at 13,000 rpm for 2 min. Aliquots of cell lysates (20 μl) containing equal amounts of protein (20-30 μg) were placed into wells of an opaque black 96-well microplate. An equal volume of luciferase substrate was added to all samples, and luminescence was measured in a microplate luminometer. The value of luciferase activity was normalized to transfection efficiency monitored by the co-transfected β-galactosidase expression vector.

### NF-κB DNA Binding Assays

Nuclear protein binding to a consensus NF-κB oligonucleotide was determined using an enzyme-linked immunosorbent assay-based kit (TransAm p65, Active Motif). Absorbance was read at 450 nm.

### Subcellular fractionation

Reh cells were resuspended in buffer A (10 mM Tris-HCl [pH 7.5], 10 mM NaCl, 3 mM MgCl_2_, 0.1 mM PMSF, 1 mM DTT. NP-40 (0.05%) was added, and the cells were incubated for 20 min on ice. The lysates were centrifuged for 5 min at 200 × *g *at 4 °C, and the supernatant collected (cytosolic fraction). The nuclear fraction was obtained by sonication of the pellet in buffer A.

### Immunoblot analysis

For immunoblot analysis, cells were lysed in radioimmunoprecipitation buffer (RIPA; 50 mM Tris-HCl [pH 7.4], 150 mM NaCl, 1% NP-40, 50 mM NaF, 10 mM β-glycerophosphate, 0.1% SDS, 0.5% EDTA, 1 mM Na_3_VO_4_, 0.2 mM PMSF, 10 μg/ml leupeptin, 0.5% aprotinin). Equal amounts of protein were separated on a 7.5% (for IKKα/β) and 10% (for other proteins) SDS-PAGE. After transfer to a nitrocellulose membrane (GE Healthcare), proteins were detected using appropriate primary antibodies and the enhanced chemiluminescence detection system (ECL Plus, Amersham Biosciences).

### Statistical analysis

SPSS 14.0.2 for Windows was used to perform statistical analysis. The paired sample *t *test was used to test significance in cell line experiments, whereas the Wilcoxon signed-rank test was applied to experiments with mice splenocytes. Specific cell death was calculated using the following equation: (% experimental cell death in the ionizing (IR)-treated sample - % spontaneous cell death in the absence of IR)/(100% - % spontaneous cell death in the absence of IR) × 100. In all figures, histograms show mean values of the indicated number of experiments with error bars corresponding to SEM values.

## Competing interests

The authors declare that they have no competing interests.

## Authors' contributions

MMK designed the research, performed experiments, analyzed data, and wrote the paper; EHN performed experiments, analyzed data and helped with data review; HC contributed vital reagents and helped with data review; HKB designed the research, analyzed data, and wrote the paper; SN provided the concept, designed the research, performed experiments, analyzed data and wrote the paper. All authors read and approved the final manuscript.
